# Measuring cardiac changes using electrical impedance during delayed cord clamping: a feasibility trial

**DOI:** 10.1186/s40748-015-0016-3

**Published:** 2015-05-22

**Authors:** Anup C Katheria, Madeline Wozniak, David Harari, Kathy Arnell, Deborah Petruzzelli, Neil N Finer

**Affiliations:** Neonatal Research Institute, Sharp Mary Birch Hospital for Women and Newborns, 3003 Health Center Drive, San Diego, CA 92123 USA; Sharp Rees-Stealy Medical Group, San Diego, CA USA

**Keywords:** Delayed cord clamping, Transitional circulation, Hemodynamics, Cardiac output

## Abstract

**Background:**

To date no study has attempted to continuously evaluate changes in hemodynamics during delayed cord clamping in humans. We aimed to demonstrate 1. the feasibility of measurements of hemodynamics during delayed cord clamping and 2. to describe the changes that occur over each minute.

**Results:**

After vaginal delivery, term infants (37^0^-41^6^ weeks) were placed on a Life Start® bed 10–20 cm below the placenta. Transcutaneous sensors were placed on the neck and chest to determine heart rate, stroke volume and cardiac output at each beat. Once a signal was obtained, first 5 values (taken every beat) were averaged and the percent change for each subject from baseline was calculated. 20 infants were enrolled and all had a reliable signal obtained from transcutaneous sensors and had a delay in cord clamping for about 5 minutes. Cardiac output increased from 2 to 5 minutes of life (p = 0.008). For every minute of life the cord was kept unclamped, the stroke volume increased 13.1% ± 12.3 (p = 0.0001) and cardiac output increased 12.6% ± 6.3 from baseline (p < 0.0001). While the majority of infants continued to have an increase in cardiac output at 5 minutes of life, 7/20 infants reached their peak cardiac output at 188 ± 41 seconds of life.

**Conclusions:**

This study demonstrates that hemodynamic measures could be successfully obtained during the first five minutes of birth and while a newborn was receiving delayed cord clamping. This study also provides reference values for changes in cardiac output and stroke volume in well term infants during delayed cord clamping.

**Trial registration:**

Clinical Trials.gov NCT02195037 Registered 17 July 2014

## Background

Traditionally a physiological transition at birth has been reserved for a small number of healthy babies cared for by midwives outside hospital. The tradition of early cord clamping at birth was introduced worldwide as part of active management of the third stage of labor (AMTL) to reduce the risk of post-partum hemorrhage. Originally introduced for the benefit of the mother without consideration of the effect on the neonate, the intervention has now been shown to have no benefit to the mother and has been removed as part of AMTL by all major health organizations including the WHO [1,2]. Early cord clamping has been shown to interfere with the physiological redistribution of blood at birth and to result in a degree of hypovolaemia and subsequent iron deficiency and anemia in the neonate [3,4]. Delayed cord clamping (DCC) allows the infant to receive blood that would otherwise remain in the placenta with immediate cord clamping. DCC increases hemoglobin levels after birth, [5] increases iron stores for up to four months and reduces childhood anemia [6]. In premature infants DCC has been shown to improve transition at birth [7]. Recently, the American College of Obstetricians and Gynecologists (ACOG) suggested a delay of 30–60 seconds after birth for premature infants mainly due to the reduction in intraventricular hemorrhage (IVH) [8]. However, the mechanism by which DCC may reduce IVH has not been established, and delayed cord clamping does not reduce severe IVH [9]. In term infants who do not require resuscitation the shift in practice is to delay the clamping of the umbilical cord for 5 minutes or until the cord stops pulsating [10].

A previous study investigated cardiac output of term infants delivered via cesarean section after a 30–60 second delay in cord clamping. Echocardiograms were taken 2, 5, and 10 minutes after birth. The investigators demonstrated an increase in cardiac output at 5 and 10 minutes compared to 2 minutes of life. These scans occurred after the cord was clamped [11]. A recent animal study documented the physiological changes at birth with delayed cord clamping comparing ventilation during delayed transfusion with immediate clamping in premature lambs [12]. The authors showed that DCC in this model with ventilator support reduced the large variability in heart rate, arterial pressures and pulmonary blood flow compared to immediate cord clamping without ventilator support. However in this complex model, the lambs were intubated immediately with their lung fluid drained prior to ventilation. To date no study has attempted to continuously evaluate changes in hemodynamics during delayed cord clamping in humans. We aimed to demonstrate 1. the feasibility of measurements of hemodynamics during delayed cord clamping and 2. to describe the changes that occur over each minute.

## Methods

Pregnant mothers with anticipated vaginal birth of term infants (37 + 0 to 41 + 6 weeks gestational age), were approached from August 2014 to September 2014 at Sharp Mary Birch Hospital for Women and Newborns. Multiples or fetus with presumed fetal anomalies were excluded. The study was approved by the Institutional Review Board at Sharp Mary Birch Hospital for Women and Newborns. Informed written consent was obtained prior to delivery. If at delivery, in the opinion of the delivering obstetrician, the infant had any maternal or neonatal indications requiring immediate cord clamping, the infant was excluded. The infants all breathed spontaneously within the first seconds of birth.

**Figure 1:**
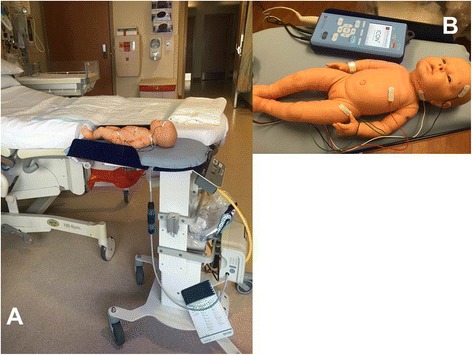
Demonstration of the Life Start Bed. **(A)** Bed placement in delivery room **(B)** ICON monitor and leads applied to above the ear, side of the neck, level of the xiphoid, and inguinal canal.

Immediately upon delivery the infant was placed on a LifeStart Neonatal Resuscitation bed (Inditherm, Rotherham, United Kingdom). The LifeStart bed was placed so the infant was 10–20 cm below the level mothers vaginal introitus (Panel A, Figure 1). The infant was dried and placed on the LifeStart trolley (heated to 39.0 C). All infants had four ICON (EC: Electrical Cardiometry, ICON device, Cardiotronic, La Jolla, CA USA) sensors placed on the left side of their head (above the ear), over the left side of their neck, over the left axilla (at the level of the nipple) and over the left inguinal canal. (Figure 1, Panel B) EC estimates cardiac output, stroke volume, and other hemodynamic parameters by sending a low amplitude, high frequency current through the body and measuring the resulting change in voltage across the thorax. This device can calculate changes in electrical conductivity to aortic blood flow at each contraction allowing a calculation of stroke volume at each cardiac beat [13]. The start time of the ICON monitor, the time of cord pulsation cessation, and the time of cord clamping were recorded by the research team using the Apgar timer. The obstetricians would periodically check (every 30–60 seconds) to determine whether cord had ceased pulsation and this was recorded by the research team. The obstetricians were informed when five minutes had passed and then the obstetrician clamped the cord. Monitoring was discontinued after the cord was cut. The infant was then given to the mother.

Relevant maternal and neonatal medical information was collected from the electronic medical record. In healthy term infants, the standard of care at Sharp Mary Birch is to take a transcutaneous bilirubin level from the infant before discharge, typically taken 24 hours after birth. This was collected as a safety measure. Any additional bilirubin and hemoglobin values were collected if available as per the infant’s hospital course. The highest bilirubin levels per the infant’s hospital course are reported. On average the transcutaneous bilirubin level was collected on day of life two, and the total bilirubin level on day of life three. Serial measurements of heart rate, cardiac output, stroke volume were obtained once leads were placed. Once a signal was obtained, first 5 samples (which are obtained every beat) were averaged to determine the subject’s initial output. All subsequent values were then averaged over each minute and compared to the initial output. For example, the first minute data were averaged from the time the signal was picked up (average 80 seconds) and until 119 seconds of life and the rest were based on 60 seconds i.e. 120 – 159 seconds. Normally distributed continuous outcome variables were analyzed within dependent samples *T*-Test and non-normal continuous outcome variables were analyzed with the Mann–Whitney *U* test. A one way repeated measured analysis of variance (ANOVA) was conducted to evaluate the null hypothesis that there was no change in each measure from baseline. Significance was set at a p value less than 0.05. Demographic data are presented as numbers and proportions.

## Results

**Figure 2:**
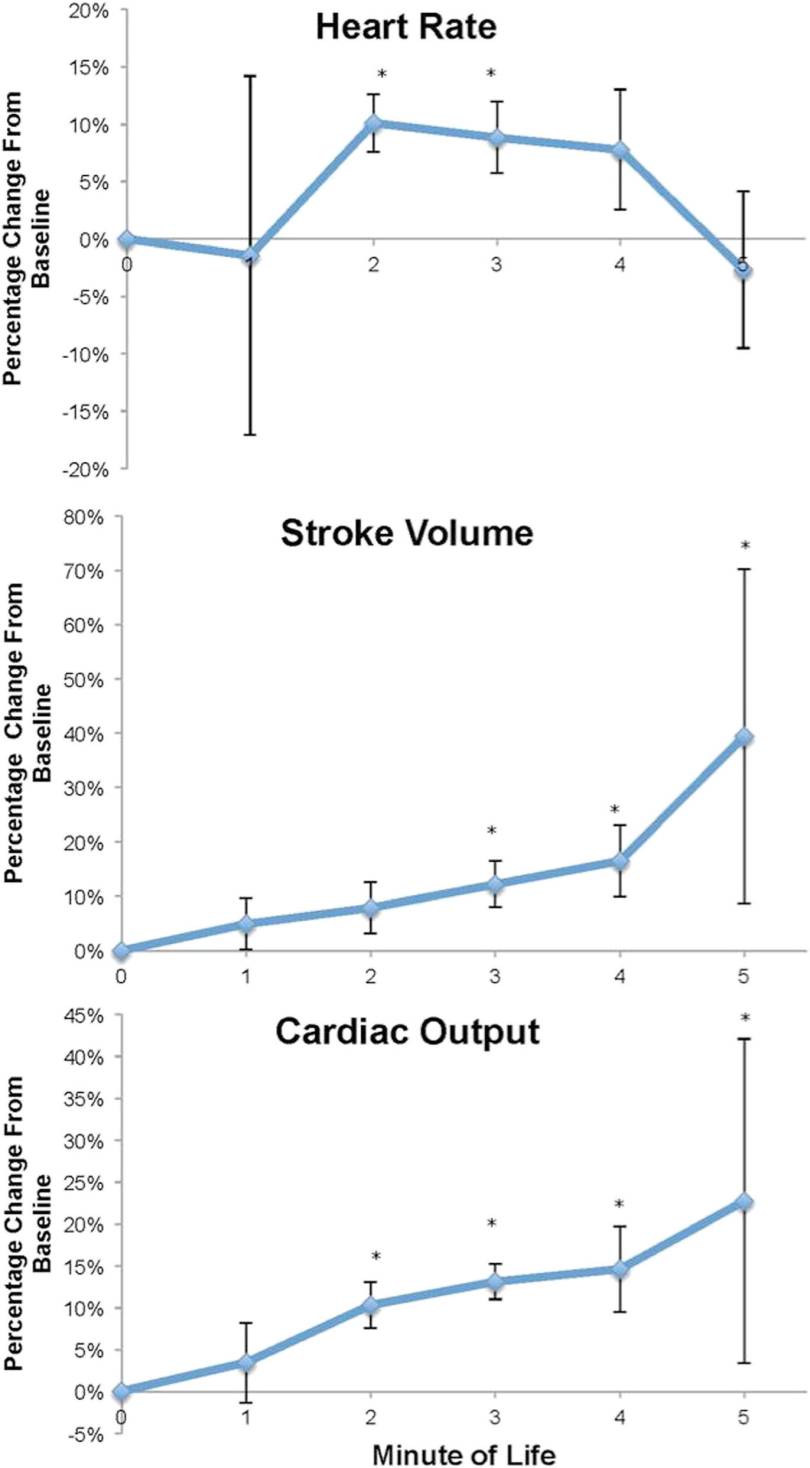
Percent change in heart rate, stroke volume, and cardiac output during delayed cord clamping over first 5 minutes. * p < 0.05.

Twenty newborns received the five minutes of delayed cord clamping (Table 1). Infants had a functioning ICON signal at 89 seconds (median, IQR [83,116]). There was no data in the first minute, and only half of the subjects had data at 1 minute. Heart rate did not significantly change over 5 minutes (Figure 1, ANOVA, p = 0.063). Stroke volume increased from 2 to 5 minutes of life (ANOVA, p = 0.016) of life (Figure 2). For every minute the cord was kept unclamped the stroke volume increased by an average of 13.1 ± 12.3 percent (p = 0.0001, compared to baseline). Cardiac output increased from 2 to 5 minutes of life (Figure 2, ANOVA, p = 0.008). For every minute the cord was unclamped the cardiac output increased by an average of 12.6 ± 6.3% (p < 0.001, compared to baseline). While the majority of infants continued to have an increase in cardiac output at 5 minutes of life, 7/20 infants reached their peak cardiac output at 188 ± 41 seconds of life. No infant had reached their peak cardiac output until at least 2 minutes of life. Values at each time point (1 – 5 minutes of life) after baseline was established for heart rate, stroke volume, and cardiac output are shown in Table 2.

**Table 1:** **Demographics**

	**Mean(SD)**
**Gestational Age (Weeks)**	39 (1)
**1 minute Median Apgar [(1 min IQR])**	9 [8 , 9]
**5 Minute Apgar [(5 min IQR])**	9 [9 , 9]
**First Axillary Temperature (°C)**	36.9 (0.2)
**Transcutaneous Bilirubin (mg/dL, n = 18)**	6.39 (4.41)
**Total Bilirubin (mg/dL, n = 13)**	8.73 (3.45)
**Hemoglobin (g/dL, n = 4)**	20.1 (2.0)
**Time Until Cord Clamped and Cut (s)**	318 (32)
**Time Until Cord Stopped Pulsating (s)**	199 (76)

Data presented as Mean (SD) unless indicated. Data on all 20 infants unless indicated.

**Table 2:** **Values of heart rate (HR), stroke volume (SV), and cardiac output (CO) in the first 5 minutes of life**

	**1 Minute**	**2 Minutes**	**3 Minutes**	**4 Minutes**	**5 Minutes**
**HR (bpm)**	175.9 (15.3)	170.7 (20.4)	170.5 (18.0)	166.7 (17.3)	168.2 (20.0)
**SV (mL)**	3.98 (1.4)	4.31 (1.4)	4.12 (1.7)	4.24 (1.8)	4.69 (1.6)
**CO (mL/kg/min)**	162 (27)	179 (19)	194 (19)	196 (19)	219 (22)

Data presented as Mean (SD) unless indicated.

The mean time to cord pulsation cessation was 199 ± 76 seconds, while four infant’s cords were still pulsating at five minutes. Infants who had cord pulsation after 3 minutes had a greater percent increase in CO in minute to minute comparisons during the delayed cord clamping compared to early cord pulsation cessation infants (<180 seconds) (1 minute p = 0.04; 2 minutes p = 0.02; 3 minutes p < 0.001; 4 minutes p < 0.001; 5 minutes p < 0.001),

## Discussion

This is the first study to demonstrate the temporal cardiovascular changes that occur during delayed cord clamping in human infants. Continuous measurements performed by EC demonstrated an improvement in stroke volume and cardiac output over the first five minutes of life, which was well after the cessation of umbilical artery pulsation.

Recently van Vorden et al. demonstrated in a single cohort of term infants delivered by Cesarean section who received a 30–60 second delay in umbilical cord had increased left ventricular output between 2 and 5 minutes of birth (34 percent) which stabilized at 10 minutes [11]. They speculated that the increases in left ventricular preload were from pulmonary blood flow and increased ductal shunting. Unfortunately the determination of left ventricular output by echocardiography is subject to intra-observer variability particularly of the aortic diameter [14]. We had previously demonstrated in preterm infants that EC derived cardiac output correlated well with left ventricular output compared with right ventricular output or superior vena cava flow [15]. While these babies had a PDA at the time of the echocardiogram (performed on day 0 and 1 of life) the measures still had significant correlation suggesting that the measures may be post ductal. A similar study in full term infants also demonstrated a very low bias with EC (−4 ml/min) and excellent correlation when compared to echocardiography measured cardiac output making it an ideal unbiased tool for measuring rapid serial changes [16]. This is of particular importance in the delivery room where the footprint of ultrasound equipment becomes a challenge. This study to our knowledge was the first to evaluate the use of EC in the delivery room and demonstrate cardiac changes in vaginal delivered infants.

Low heart rates during delayed cord clamping were observed in a cohort of 109 term infants who were immediately placed skin to skin on the mother’s chest compared to another cohort of babies who received immediate clamping [17]. The authors speculated that the lower heart rate which did not reach >100 beats per minute until 3 minutes of life may be due the calming effect of skin to skin (less tachycardia), or because DCC prevents a loss in preload and stroke volume there may be less need to compensate by increasing their heart rate. While we did not see a low heart rate in our study, our study is consistent with these findings since we also did not see a change in heart rate over the first 5 minutes despite a change in cardiac output and stroke volume.

Our study aimed to demonstrate the hemodynamic changes during delayed cord clamping using EC. Delayed cord clamping has been shown to improve measures of systemic blood flow by echocardiography compared to immediate cord clamping for several days after birth [18]. While delayed cord clamping has beneficial effects on preterm and term newborn infants, the optimal duration of delayed cord clamping has not been established [19]. The use of additional monitoring such as EC may be able to help clinicians establish when adequate cardiac output has occurred by providing a signal as to the optimal time for cord clamping. The current practice of waiting until a pre-specified time may not be ideal compared to ensuring that an adequate cardiac output has occurred before cord clamping has occurred. As seen in our study, several infants may reach their peak cardiac output before 5 minutes, while others may need more time. Performing ultrasound Doppler of the cord may be another method to determine the correct amount of transfusion required for each newborn. Recently an abstract at the European Pediatrics Academic Society meeting, which utilized ultrasound Doppler of the cord, demonstrated that only 33% of term infants still had venous flow at the time of cord clamping (range 2:56–9:15) [20]. Either of these devices at delivery may better help clinicians particularly in infants that require resuscitation.

There are a number of limitations to this study. Since this was a feasibility study on healthy term infants our sample size and population is too small to make any conclusions about the clinical impact of delayed cord clamping. Our study demonstrates the changes in SV and CO during delayed cord clamping. We did not measure the volume of blood in this study, but presumably all of these infants had improvements in cardiac output and stroke volume related to increased volume. Our results show that there is a continuing increase in SV and CO independent of HR for the first 5 minutes in most infants. Similar to other studies of pulse oximetry and heart rate monitoring we could not obtain a signal from our EC sensors before the first minute of life.

A limitation of our study is that we did not obtain a comparison group of infants receiving early or immediate cord clamping, since the aim of our study was to determine the infant’s physiological changes occurring during delayed cord clamping. In our center delayed cord clamping is practiced by the majority of our obstetricians and often requested by parents. We did not feel it would be ethical to randomize newborns to receive a treatment that would change our accepted practice or be against parental wishes. In addition we did not obtain weights of the infants to compare the amount of placental transfusion. Lastly to limit the amount of monitoring during the bonding period we also did not continue monitoring past 5 minutes. We believe this study provides pilot important information and future studies will build on our data to determine the normal range of hemodynamics of the neonate immediately after birth.

## Conclusion

In conclusion, this study demonstrates that hemodynamic measures could be successfully obtained during delayed cord clamping. This study also provides reference values for changes in cardiac output and stroke volume during delayed cord clamping in well term infants.

## Abbreviations

DCC: Delayed cord clamping
ACOG: American College of Obstetricians and Gynecologists
IVH: Intraventricular hemorrhage
EC: Electrical cardiometry
ANOVA: Analysis of variance
